# Identifying Risk Factors for Dental Neglect in Children Who Failed to Complete Their Dental Surgery Appointments in Northeast Ohio: A Retrospective Study

**DOI:** 10.3390/children12060670

**Published:** 2025-05-23

**Authors:** Ying An, Margaret Ferretti, Lindsey Jones, Eilish Welsh, Justin McCray, Seungchan Kim, Maya Thompson, Gerald Ferretti

**Affiliations:** 1 Department of Pediatric Dentistry, School of Dental Medicine, Case Western Reserve University, Cleveland, OH 44106, USA; ying.an@case.edu (Y.A.); mef18@case.edu (M.F.); lgj19@case.edu (L.J.); jrm283@case.edu (J.M.); sxk1731@case.edu (S.K.); maya.thompson@case.edu (M.T.); 2 University Hospitals Rainbow Babies and Children Hospital, Cleveland, OH 44106, USA

**Keywords:** dental neglect, pediatric dentistry, caries, intravenous sedation, general anesthesia, barriers to care

## Abstract

**Background/Objectives:** For dental providers, it can be difficult to distinguish dental neglect from legitimate barriers to care, preventing the completion of dental treatment for pediatric patients. The aim of this study was to utilize caretakers’ self-reported reasons for missing their child’s appointments to identify barriers to treatment completion versus dental neglect. **Methods:** The treatment setting was oral rehabilitation under deep or intravenous sedation (IV) or general anesthesia (GA). After the responses were examined, patients were categorized into one of four groups: Low-Risk, Treatment Completed; Low-Risk, Treatment Not Completed; High-Risk, Treatment Completed; High-Risk, Treatment Not Completed. Low- or high-risk classifications were determined based on whether the reported rationale was a temporary hindrance that could be overcome through additional effort or assistance, or whether it was because the parent or guardian did not follow through with the recommended instructions and treatment. A chi-square test was completed. **Results:** A total of 602 IV charts and 1, 296 GA charts were reviewed for this study. For both IV and GA settings, the proposed low- and high-risk factors for dental neglect were statistically significant (IV: *p* = 0.000442; GA: *p* < 0.00001). **Conclusions:** The patients whose appointments were not completed for reasons deemed high risk for dental neglect had a higher risk of having uncompleted treatment. These patients should be closely followed due to a higher risk of dental neglect.

## 1. Introduction

Dental caries are the most common chronic disease in children of the United States, and when left untreated, can lead to dental neglect [1]. The presence of carious lesions alone is not grounds for dental neglect, but rather, according to the American Academy of Pediatric Dentistry (AAPD), it is the “willful failure of parent or guardian to seek and follow through with treatment necessary to ensure a level of oral health essential to adequate function and freedom from pain and infection” [2,3]. The World Health Organization has expanded upon this further, saying that “neglect must be distinguished from circumstances from poverty, implying that neglect can only occur in cases where reasonable resources are available to the family or caregiver” [2]. Poverty, poor health literacy, and isolation amplify the effects of neglect [4]. According to Loochtan, Bross, and Domoto, the aforementioned factors can and should be forgiven when considering a potential case. However, when these significant barriers to treatment are eliminated and the pathology is clearly explained, it is expected that care be completed in a timely manner [4]. If the prescribed treatment for the child is not achieved, dental neglect can lead to severe consequences to the child’s overall health and well-being.

The effects from the deterioration of a child’s oral health can disseminate into all aspects of his or her life. The consequences include pain [2], sleep deprivation [2,5,6], trouble with eating [6], increased teasing from classmates [5], and decreased performance at school [2,6]. Untreated dental necrosis may lead to infection that remains localized or may spread, resulting in cellulitis [7]. Facial cellulitis in children is extremely serious due to its ability to rapidly transmit into deep spaces, leading to an inflammatory response that could result in septic shock, multiple organ failure, and even death [8].

Due to the precarious nature of untreated dental caries, dental providers are mandated reporters of dental neglect [1]. However, despite the gravity of this matter, the literature suggests that there is a low rate of reporting to authorities when child neglect is ascertained [1,9]. In Massachusetts, it was determined that only 20% of suspected dental neglect cases were reported [1]. This is not due to dentists overlooking these cases, as one study showed that 81% of dentists reported examining children with neglected dentitions once or more per week, and 59.9% said this occurred daily [10]. If dental neglect is this prevalent in the dental office, why is reporting to appropriate authorities so low? Studies show that dental providers may fear possible repercussions for reporting, such as damaging their ability to gain new patients, future involvement in civil suits, and financial cost [1]. Additionally, the United States’ jurisdiction laws on dental neglect are inconsistent [11]. Despite the fact that all 51 jurisdictions have laws that define child abuse and neglect, only New Jersey, New York, Oklahoma, Oregon, Pennsylvania, Utah, Virginia, and Wisconsin specify dental neglect in their definitions; none of the 51 jurisdictions use the AAPD’s definition [11].

Aside from inconsistent legislature among 50 states, there are additional barriers in caregivers seeking services for their children’s oral health. Children in the United States are less likely to have dental insurance than medical insurance, with the CDC revealing that there are 2.6 times as many children with no dental insurance as those with no health insurance [12]. This equates to 26 million youth in the United States without dental coverage [12]. Additionally, the caregiver’s attitude towards dental treatment can be a barrier for pediatric treatment [13]. Many adults only seek dental care for emergency treatment and do not return to restore their dentition to complete oral health. This approach may also be adopted for their children [13]. It has also been determined that many parents believe dental care is secondary to medical care and are less likely to follow-up with preventive dental services than medical services for their children [13]. Finally, caregivers of lower socioeconomic standing have reported that their lives are too busy and complicated to follow-up with their children’s oral health care, stating factors such as length of time for appointments, transportation, difficulty coordinating with employment, and finding childcare for siblings [14].

The purpose of this study is to aid dental practitioners in identifying potential dental neglect cases by analyzing the reasons that parents or legal guardians gave after missing their child’s sedation or anesthesia appointment. We investigated dental neglect among patients to whom dental treatment under general anesthesia (GA), deep sedation, or intravenous sedation (IV) was recommended because of an increased vulnerability to morbidity when treatment was not completed. At University Hospitals Rainbow Babies and Children’s Hospital, located in Cleveland, OH, USA, patients were recommended for dental treatment under GA or IV for the following reasons: (1) younger age and/or special health care needs [15]; (2) more likely to have an extensive and/or urgent need of dental treatment that cannot be completed in an outpatient setting; and (3) limited access to dental care, preventing the need for multiple appointments. These patients are at higher risk of odontogenic pain and infection. They are not able to advocate for themselves, relying on a parent or guardian to do so on their behalf.

## 2. Materials and Methods

This study was approved by the Institutional Review Board (IRB20200317) of University Hospitals. The study was a retrospective chart review of pediatric patients that came to University Hospitals Rainbow Ahuja Center for Women and Children Dental Clinic. All patients that were recommended for complete comprehensive dental care under GA from 1 September 2018 to 31 August 2019 or under IV from 1 September 2018 to 31 August 2021 were included in the study. Eligible patients’ data were obtained from our electronic dental practice management software, Dentrix Enterprise^TM^ version 8.0.7.485, by using four in-office codes: IV Recommendation, OR Recommendation, IV Reservation, and OR Reservation. The data consisted of patients’ demographic information (gender and age), whether the sedation appointments were completed, any rescheduled appointment(s), and the documented reasons for a failed appointment.

Self-reported reasons for failed appointments were categorized into two groups of dental neglect potential (low risk vs. high risk). The risk level of dental neglect was determined by whether self-reported reasons could be overcome by reasonable additional assistance and effort, such as rescheduling the appointment, financial assistance, and Medicaid transportation arrangement. The low-risk indicators are those that can be overcome by additional effort from parents or providers, which included the following: family emergency, patient illness, cancelation due to COVID-19 for patients not lost to follow-up (see exclusions), lack of insurance coverage [12], personal conflicts [13], transportation issues [14], treatment completed or to be completed in a different setting (chairside or different clinics), and other. The high-risk indicators were those that suggested the parents or guardians willingly chose not to follow through with the surgical treatments and/or ignored the pre-surgical instructions, regardless of being informed of the necessity of treatment and potential severe consequences for not following through [1,2,3]. At University Hospitals, during the preoperative assessment, instructions and dental education were given to all families one month, one week, and one day prior to the surgery date, which included arrival time, NPO (fasting) instructions, and the importance of abiding to these instructions. Deviations from these instructions included not keeping to the NPO guidelines, not showing up on time for surgery, not answering the provider’s instruction calls, and not completing necessary pre-surgery medical appointments, ultimately leading to treatment not being completed. Therefore, these self-reported reasons are considered as higher risk of dental neglect. High-risk indicators included the following: canceled without reason in less than 24 h, failure to commit, no-show on the day of surgery, NPO violation, required pre-surgery medical appointment not completed, late for surgery start time leading to a canceled appointment, and provider unable to confirm the surgery. The detailed definition of each category is shown in Table 1.

**Figure 1:**
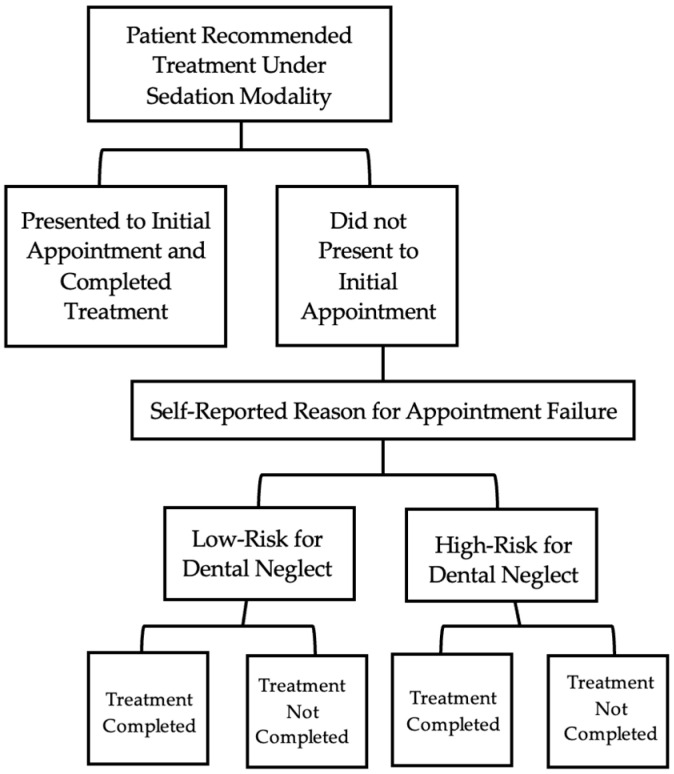
Outline of patient chart categorization.

The chart of each patient was examined to determine if treatment was completed after the initial failed appointment. Patients previously categorized in either the low- or high-risk groups were then sorted based on whether treatment was completed and compiled into four categories: Low-Risk, Treatment Completed; Low-Risk, Treatment Not Completed; High-Risk, Treatment Completed; High-Risk, Treatment Not Completed. The general categorization of patients’ charts is outlined in Figure 1.

Exclusions: The COVID-19 pandemic occurred during the study. As a result, patients lost to follow-up due to clinic cancelations were excluded, as this was an unforeseen barrier to treatment. However, patients whose appointment was initially canceled due to COVID-19 restrictions, but completed treatment, were included in the study.

Statistics: Demographic data such as gender and age were collected. Descriptive statistics using frequencies and percentages were used to describe the number of patients for whom treatment under IV and GA was recommended and to determine the number of patients who did or did not receive treatment. Descriptive statistics were also used to describe the self-reported reasons a patient did not present for treatment. A chi-square test for independence was performed to determine whether the proposed low- and high-risk indicators were indicative of patients at substantial risk for dental neglect. The patients who never completed the recommended IV/GA appointments (Low-Risk, Treatment Not Completed; High-Risk, Treatment Not Completed) were compared by using chi-square analysis. SPSS 28.0 was used for statistical analysis and all tests utilized a 0.05 level of significance.

## 3. Results

### 3.1. IV

A total of 602 charts were reviewed where the patient was referred to complete dental treatment under intravenous sedation from 1 September 2018 to 31 August 2021. The mean age of patients was 5.8 ± 2.6 (standard deviation, SD) years old. The age range was from 1 to 17 years old. Out of 602 patients, 290 of them were female and 312 of them were male. A total of 436 of these patients attended their appointment at the originally scheduled time, while 166 (27.57%) did not. Of these 166 patients, 7 were excluded from the study due to COVID-19 clinic cancelations and being lost to follow-up, making an adjusted total of patients who did not receive treatment under IV as N = 159.

Of the initial 159 patients who did not show up to their first IV appointment, the three most common reasons that parents reported failure to attend were (1) no-show (n = 43, 27.04%), (2) failure to commit (n = 23, 14.47%), and (3) patient illness (n = 19, 11.95%). Table 2 further analyzes the patients based on both their risk category and whether treatment was completed. A total of 38 patients whose reasons were considered high risk completed treatment, while 59 did not. A total of 42 patients whose reasons were considered low risk completed treatment, and 20 did not. These values were analyzed using chi-square test, determining a significant correlation (*p* = 0.00042) between high-risk indicators and dental neglect in comparison to low-risk indicators.

### 3.2. General Anesthesia

A total of 1296 patient charts were reviewed where the patients were referred to complete dental treatment under general anesthesia from 1 September 2018 to 31 August 2019. The mean age of patients was 7.9 ± 6.1 (SD) years old. The age range was from 1 to 39 years old. Out of 1296 patients, 582 of them were female and 714 of them were male. A total of 868 of these patients attended their appointment at the originally scheduled time, while 428 (32.87%) did not. A total of 7 of the 428 patients were excluded from the study due to COVID-19 clinic cancelations and being lost to follow-up, making an adjusted total of patients who did not receive treatment under GA as N = 421.

Of the initial 421 patients who did not show up to their first appointment, three of the most common reasons parents reported a failure to attend were failure to commit to the rescheduled appointment (n = 94, 22.33%), patient illness (n = 77, 18.29%), and required pre-surgical medical appointment not completed (n = 62, 14.73%). Table 3 further analyzes the patients based on both their risk category and whether treatment was completed. Overall, 107 patients were high-risk and completed treatment, 157 were high-risk and did not complete treatment, 107 were low-risk and completed treatment, and 50 were low-risk and did not complete treatment. These values were analyzed using chi-square test, determining a significant correlation (*p* < 0.00001) between high-risk indicators and dental neglect in comparison to low-risk indicators.

## 4. Discussion

This study aimed to determine which self-reported reasons parents and guardians gave for missing their child’s procedure were indicators of dental neglect. These responses were recorded and categorized based on whether the barriers could be overcome with reasonable additional effort (low risk), or if they suggested that the parents or guardians chose to neither seek, nor follow through, with recommended treatment though access to care was not a concern (high risk). Low-risk reasons also included obstacles that were deemed temporary (i.e., illness, emergencies) or were due to a reliance on an outside source (i.e., transportation, insurance coverage, etc.).

Most patients (more than 98%) in this study were of a low socioeconomic background and had state-funded dental insurance. This patient population is highly homogenous regarding the financial status. This means that almost all of the study subjects are faced with barriers to dental care that include difficulty in finding providers that accept state insurance, unreliable transportation, excessive wait times, demeaning interactions with dentists and staff due to perceived judgment from Medicaid coverage, and balancing daily responsibilities to make appointments [16]. It is worth noting that our study determined that the self-reported reasons stemming from state coverage insurance, low socioeconomic background, and balancing daily obstacles were determined to be low risk for dental neglect. As providers, we must be aware and understanding of these hindrances this patient population faces, providing additional support and opportunities for them to complete their dental treatment.

When the barriers to care (low-risk factors) are alleviated from the patients with low socioeconomic status through additional support, the treatment is more likely to be completed and therefore these factors should not qualify the patients as having an increased risk of dental neglect. However, when the same support was given to those patients with the high-risk factors, they were more likely to not complete treatment and therefore have a higher risk of dental neglect. For example, it was observed that in both IV and GA settings, two of the most common reasons for appointment failure were the same: patient illness and failure to commit to scheduled appointment. Patient illness was concluded to be low-risk for dental neglect; for IV and GA combined, of the 96 patients who did not show up to the original appointment due to illness, 81 patients completed their treatment by the end of the study. On the contrary, failure to commit to an initial or rescheduled appointment was deemed to be high-risk for dental neglect in both treatment settings. Out of 117 combined patients, 108 patients never completed their recommended treatment. These values highlight the importance of allowing patients who are ill additional chances to attend their sedation appointments, as treatment will presumably be completed. Additionally, these numbers reflect on the importance of follow-up with patients whose caregivers fail to commit to an appointment, as this is strongly indicative of dental neglect.

The most frequently reported cause for missing an IV appointment was No-Show, a high-risk indicator of dental neglect. For GA, it was Failure to Commit, which is considered high-risk for dental neglect. One study, conducted at a Federally Qualified Health Center (FQHC) in Dayton, Ohio, reported that adopting a no-show and termination system is statistically significant for decreasing no-show rates [17]. For the first two no-shows, letters were sent to the patient’s address explaining the consequences of missing a third time. After a third no-show, the family was dismissed from the practice. To be reinstated into the practice, the parents or guardians were required to attend an hour of class educating them on the importance of committing and following through with these appointments [17]. This system could be adopted by hospitals for patients’ families exhibiting behavior indicative of high-risk for dental neglect, allowing the caregivers a chance to be educated on dental neglect and the risk it poses to their children, and a chance to receive treatment before potential legal involvement [11]. If intervention is appropriate, this study’s information can also be used to establish a protocol to aid providers in reporting to Child Protective Services. As mentioned, dentists are hesitant to report dental neglect due to inconsistent jurisdiction laws and potential negative repercussions. Therefore, the creation of a protocol would be of value [1,10]. Based on the information obtained from our study, we can establish a reporting protocol for CPS by flagging patients who consistently do not show up for sedation appointments, with unscheduled sedation appointment and failure to attend required medical appointment prior to surgery date.

There are several study limitations. (1) The data collected were from one hospital-affiliated pediatric dental residency program in the Greater Cleveland, Ohio area, and were therefore limited to patients’ demographic information in this specific geographic location. Thus, the information may not be applicable to other demographics within the state of Ohio or throughout the United States. (2) The study relied on parental self-reported data. This could introduce the opportunity for the misrepresentation of data if, for example, a parent gave a low-risk indicator, such as patient illness, but the real reason was an NPO violation. Additionally, the reverse could happen; for example, a patient does not show up on the day of the appointment without warning, when in reality the child is sick. (3) The potential impact of the COVID-19 pandemic on caretakers seeking dental treatment for their children remained unclear. While no parents or guardians explicitly stated an apprehension of the COVID-19 pandemic as the reasoning for not completing their child’s procedure, the potential effect it could have had on the study cannot be ignored. Very little information has been published regarding the effect of COVID-19 on parents’ decision to schedule non-emergent pediatric dental surgeries, or whether dental neglect increased during this time. One study, however, did determine that 66.6% of parents would only seek emergent dental care for their children and 83% did not seek dental care at all during the pandemic [18]. (4) There are alternative explanations to the reasons that the treatments were not completed. These could be, for example, parents changing phone numbers, moving out of town, or the treatment being completed elsewhere. This still presents as a high-risk behavior because the parents failed to communicate any possible alternative reasons to the dental service.

The data generated from this project were gathered in hopes of creating a future protocol to aid dentists in identifying and preventing pediatric dental neglect when patients are referred to treatment under intravenous sedation or general anesthesia. It also was created to encourage purposeful follow-up for patients at high-risk of dental neglect, increasing their chances for treatment completion.

## 5. Conclusions

1. In both IV and GA settings, patients whose appointments were not completed for reasons deemed as high risk for dental neglect were more likely to have uncompleted treatment.
2. In the GA setting, the top three high-risk factors for not completing treatment were as follows: (1) failure to commit to schedule the appointment; (2) required medical appointment not completed; (3) no-show on the day of surgery.
3. In the IV setting, the top two high-risk factors for not completed treatment were: (1) no-show on the day of surgery; (2) failure to commit to schedule the appointment.
4. Because of the findings from this retrospective review, the authors recommend that a future study prospectively evaluating a clinical protocol to report dental neglect cases based on identified high-risk factors is needed.

## Figures and Tables

**Table 1:** Definitions of low- and high-risk indicators of dental neglect based on self-reported reasons for appointment failure.

Low-Risk Factors for Dental Neglect	
*COVID-19 Cancelation*	Patient’s appointment was canceled by the clinic due to COVID-19 restrictions
*Family Emergency*	Parent or guardian reported an urgent family matter
*Lack of Insurance Coverage*	Patient did not have active insurance at time of appointment
*Patient Illness*	Patient had an illness that warranted delay of appointment
*Personal Conflict*	Patient’s parent’s or guardian’s morals or ideology did not align with suggested treatment after further consideration
*Transportation Issues*	Patient was unable to secure transportation for appointment
*Treatment in Different Setting*	Patient completed, or is to complete, treatment in other treatment modality or facility
*Other*	Reason does not fit in aforementioned categories
High-Risk Factors for Dental Neglect	
*Canceled Without Reason < 24 h*	Guardian canceled previously confirmed appointment < 24 h before appointment without reason
*Failure to Commit*	Patients whose appointment was not scheduled after guardian agreed to suggested treatment
*No-Show on the Day of Surgery*	Patient did not arrive for appointment after appointment was confirmed
*NPO Violation*	Patient broke fasting guidelines given before surgery
*Required Medical Appointment Not Completed*	Patient required clearance visit to specialty physician or primary care physician and did not attend
*Start Time*	Patient failed to show at an appropriate time for procedure to take place
*Unable to Confirm*	Schedulers were unable to contact family to confirm appointment

**Table 2:** Assessment of self-reported factors associated with failed pediatric dental appointments using intravenous sedation (IV).

Low-Risk Factors for Dental Neglect (N = 62) (%)		
Reasons for Missing Appointments	Treatment Completed	Treatment Not Completed
*COVID-19 Cancelation*	n = 7 (11.29)	excluded
*Family Emergency*	n = 0	n = 2 (3.23)
*Lack of Insurance Coverage*	n = 3 (4.84)	n = 5 (8.06)
*Patient Illness*	n = 16 (25.81)	n = 3 (4.84)
*Personal Conflict*	n = 5 (8.06)	n = 6 (9.68)
*Transportation Issues*	n = 3 (4.84)	n = 1 (1.61)
*Treatment in Different Setting*	n = 8 (12.90)	n = 1 (1.61)
*Other*	n = 0	n = 2 (3.23)
Total	**n = 42 (67.74)**	**n = 20 (32.26)**
High-Risk Factors for Dental Neglect (N = 97) n (%)		
Reasons for Missing Appointments	Treatment Completed	Treatment Not Completed
*Canceled Without Reason <24 h*	n = 0	n = 0
*Failure to Commit*	n = 2 (2.06)	n = 21 (21.65)
*No-Show Day of Surgery*	n = 20 (20.62)	n = 23 (23.71)
*NPO Violation*	n = 8 (8.25)	n = 3 (3.09)
*Required Medical Appointment Not Completed*	n = 4 (4.12)	n = 5 (5.15)
*Start Time*	n = 4 (4.12)	n = 2 (2.06)
*Unable to Confirm*	n = 0	n = 5 (5.15)
Total	**n = 38 (39.18)**	**n = 59 (60.82)**

**Table 3:** Assessment of self-reported factors associated with failed pediatric dental appointments using general anesthesia (GA).

Low-Risk Factors for Dental Neglect (N = 157) n (%)		
Reasons for Missing Appointments	Treatment Completed	Treatment Not Completed
*COVID-19 Cancelation*	n = 1 (0.64)	excluded
*Family Emergency*	n = 1 (0.64)	n = 0
*Lack of Insurance Coverage*	n = 20 (12.74)	n = 15 (9.55)
*Patient Illness*	n = 65 (41.40)	n = 12 (7.64)
*Personal Conflict*	n = 15 (9.55)	n = 10 (6.37)
*Transportation Issues*	n = 3 (1.91)	n = 3 (1.91)
*Treatment in Different Setting*	n = 0	n = 0
*Other*	n = 2 (1.27)	n = 10 (6.37)
Total	**n = 107 (68.15)**	**n = 50 (31.85)**
High-Risk Factors for Dental Neglect (N= 264) n (%)		
Reasons for Missing Appointments	Treatment Completed	Treatment Not Completed
*Canceled Without Reason <24 h*	n = 3 (1.14)	n = 10 (3.79)
*Failure to Commit*	n = 7 (2.65)	n = 87 (32.95)
*No-Show Day of Surgery*	n = 26 (9.85)	n = 16 (6.06)
*NPO Violation*	n = 7 (2.65)	n = 3 (1.14)
*Required Medical Appointment Not Completed*	n = 34 (12.88)	n = 28 (10.61)
*Start Time*	n = 7 (2.65)	n = 0
*Unable to Confirm*	n = 23 (8.71)	n = 13 (4.92)
Total	**n = 107 (40.53)**	**n = 157 (59.47)**

## Data Availability

The datasets presented in this article are not readily available because the dataset contains patient health information which is protected by HIPPA law. University Hospitals’ IRB prohibits the sharing of the original dataset to the public.
